# Specific Recognition of Glycoproteins: Design Strategies and Application Prospects of Molecularly Imprinted Polymers

**DOI:** 10.3390/ijms27010528

**Published:** 2026-01-04

**Authors:** Ping Xie, Zi-Ying Chen, Chun-Fang Xie, Jie-Ping Fan

**Affiliations:** 1 Department of Chemical Engineering, School of Chemistry and Chemical Engineering, Nanchang University, Nanchang 330031, China; 2 Key Laboratory of Poyang Lake Ecology and Bio-Resource Utilization of Ministry of Education, Nanchang University, Nanchang 330031, China

**Keywords:** molecularly imprinted polymers, glycoproteins, specific recognition, oriented surface imprinting, epitope imprinting, post-imprinting modification

## Abstract

Glycoproteins pose significant challenges for specific recognition due to their structural complexity and microheterogeneity. Molecularly imprinted polymers (MIPs) have emerged as promising synthetic receptors, offering high stability and tailorable recognition sites. This review specifically highlights and systematically evaluates several cutting-edge design strategies tailored for glycoproteins, including oriented surface imprinting for site-accessible recognition, epitope imprinting for enhanced specificity, and post-imprinting modification for tailored functionality. The fundamental principles, technical advantages, and applications in glycoprotein detection and separation are thoroughly discussed, with a particular emphasis on a comparative analysis to guide strategy selection and how they collectively address the persistent challenges of traditional imprinting. Future perspectives highlight stimuli-responsive systems, multimodal recognition, and computational design to advance MIPs as indispensable tools in proteomics and personalized medicine. The synergistic integration of these advanced strategies within sustainable and standardized MIP systems is particularly promising for fabricating next-generation synthetic receptors with enhanced recognition capabilities.

## 1. Introduction

Glycoproteins are a class of conjugated macromolecules, comprising a protein moiety covalently attached to one or more carbohydrate chains (oligosaccharides or glycans) via glycosylation [1]. They are classified into two major categories, N-linked (e.g., immunoglobulin G, ovalbumin) and O-linked (e.g., mucins, glycophorin), based on the linkage of their carbohydrate chains to the polypeptide backbone amino acids [1]. The oligosaccharide/glycan is attached to the side-chain nitrogen of an asparagine residue for N-linked glycoproteins [2], and to the hydroxyl oxygen of a serine or threonine residue for O-linked glycoproteins [3]. These oligosaccharide/glycan chains function as a vital “molecular language”, mediating essential fundamental biological processes such as cell proliferation, cell recognition, signal transduction, and immune responses [4,5,6]. Given these critical functions, glycoproteins are closely related to human health and constitute a major class of therapeutic agents for numerous diseases [7,8]. Specifically, they serve as crucial drugs in various forms, including cytokines, monoclonal antibodies, and enzyme replacement therapies [9,10,11]. For example, the erythropoietin, interferon β, and granulocyte colony-stimulating factor (G-CSF) among cytokines [12,13]; trastuzumab, rituximab, and bevacizumab among monoclonal antibodies [14,15]; and imiglucerase, agalsidase β, and laronidase as enzyme replacement therapies [16]. Furthermore, the precise expression and structure of glycoproteins are critical for normal physiological function, making them invaluable clinical biomarkers, since alterations in their expression levels or glycosylation patterns provide highly sensitive indicators of pathological states [17,18]. In this respect, the separation and enrichment of glycoproteins from complex biological matrices with high efficacy is vitally important to their applications. However, this process faces significant challenges due to the low abundance of target glycoproteins, the extreme complexity of the sample matrices, and the inherent microheterogeneity of glycoprotein structures [19,20]. Therefore, the development of highly efficient separation and enrichment methods is fundamental toward achieving the precise detection and full utilization of glycoproteins in therapeutic and diagnostic applications.

To date, numerous strategies have been established for the recognition of glycoproteins, including specific antibodies [21], lectin affinity assays [22], hydrazide chemistry-based enrichment [23], and hydrophilic interaction liquid chromatography (HILIC) [24]. Nevertheless, these conventional approaches are often hindered by inherent drawbacks, such as cumbersome procedures, high cost, limited stability, and often insufficient selectivity, particularly when dealing with glycoprotein microheterogeneity or complex biological matrices [25,26]. In contrast, molecularly imprinted polymers (MIPs) have emerged as a promising synthetic alternative, offering distinct advantages in terms of synthetic accessibility, low cost, high physical/chemical stability, reusability, and the ability to tailor recognition sites toward specific glycoforms or protein epitopes—a level of customization difficult to achieve with natural receptors like antibodies or lectins [26,27,28]. Over the past decades, MIPs have thus gained attention not merely as a complementary tool, but as a potentially irreplaceable platform for glycoprotein recognition in scenarios demanding robustness, scalability, or specificity toward defined glycosylation variants.

**Figure 1:**
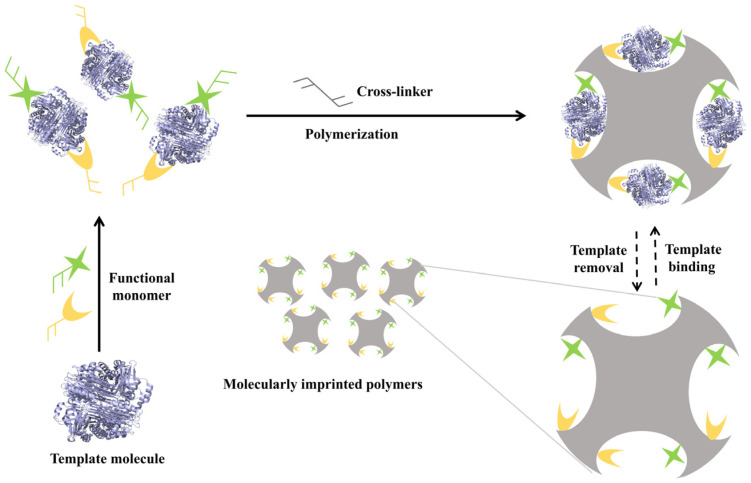
Schematic illustration of molecular imprint polymer preparation process. Reproduced with permission [29]. Copyright 2024, Elsevier.

MIPs are synthetic materials engineered with specific recognition sites through molecular imprinting technology (MIT) [27,29,30]. These sites are complementary to the target molecule in size, shape, and functional groups. The standard fabrication process involves the copolymerization of functional monomers with a cross-linker to form a rigid polymer network, of which the functional monomers are pre-assembled around a template via covalent or non-covalent interactions [27,29,31]. Subsequent template removal creates the complementary binding cavities, as illustrated in Figure 1 [29].

While early observations by Polyakov et al. [32] in 1931 hinted at the imprinting concept, the modern field of MIT was firmly established in the 1970s through the foundational work of Wulff [33,34], who developed covalent imprinting approaches, and Mosbach [35,36], who pioneered the more versatile non-covalent imprinting method for small molecules. Subsequently, Glad et al. [37] successfully extended the MIT to proteins recognition and separation in the 1980s, representing a milestone in the field of protein imprinting. Since then, the application of MIT in protein recognition has been rapidly developed [38], various MIPs toward proteins recognition with high efficiency have been constructed, especially with the advance of material science and polymerization strategies.

Previous reviews have often provided broad overviews of protein imprinting, covering key aspects such as the selection of functional monomers [39], template protein removal [40], advances in protein imprinting [38], as well as its applications in bio-enrichment [41] and biomimetic sensing platforms [42]. However, there is still a lack of dedicated, critical analysis on strategies designed for the unique challenges of glycoproteins. To address this gap, this review offers a systematic examination of several promising design strategies—oriented surface imprinting, epitope imprinting, and post-imprinting modification (PIM). Among them, oriented surface imprinting tackles inefficient template removal and mass transfer by confining sites to surfaces, epitope imprinting counters structural instability and difficult template removal by using stable epitope templates, PIM modification enhances specificity and functionality to reduce non-specific binding. Accordingly, the fundamental principles, technical advantages, and representative applications of each strategy are systematically reviewed. This integrated discussion consistently highlights their collective role in resolving the long-standing challenges, thereby providing strategic guidance for advancing MIPs in glycoprotein recognition.

## 2. Challenges in Glycoprotein Imprinting

MIPs have shown considerable promise in glycoprotein recognition, but their development and practical application face significant challenges due to the intrinsic structural complexity of glycoproteins [43]. The principal obstacles can be logically categorized into three primary areas.

(a) Structural instability and conformational heterogeneity

As a class of biomacromolecules, glycoproteins exhibit considerable conformational flexibility, rendering them susceptible to structural alterations during the imprinting process [1]. In addition, an identical polypeptide backbone can be linked to a heterogeneous population of glycan chains, resulting in inherent microheterogeneity [1]. This inherent microheterogeneity presents a fundamental barrier to generating homogeneous recognition sites with consistent binding affinity. Furthermore, the imprinting process frequently employs non-physiological conditions, such as the use of organic solvents or acidic environments for template removal, which can disrupt the native conformation of glycoproteins and ultimately compromise the biological relevance of the resulting imprinted cavities.

(b) Inefficient template removal and mass transfer limitations

Glycoproteins, due to their large molecular size and complex three-dimensional structure, are often deeply entrapped within the densely cross-linked polymer networks [27,44]. This fundamental issue makes complete template removal challenging, resulting in residual templates that permanently occupy recognition sites, diminish binding capacity, and cause false-positive signals [27,44]. Furthermore, aggressive elution using strong acids or solvents, while often necessary for deep-seated templates, risks swelling or degrading the polymer, compromising the integrity of the larger, more complex glycoprotein cavities. Consequently, the high cross-linking density essential for maintaining cavity stability, combined with the large size of the target, creates significant steric hindrance. This hindrance limits the accessibility of target glycoproteins to the cavities, leading to slow binding kinetics and reduced imprinting efficiency, which is a particular drawback for high-throughput separation or real-time analysis [27,44].

(c) Prominent non-specific binding and insufficient selectivity

The complex surface characteristics of glycoproteins, featuring both charged amino acid residues and hydrophilic carbohydrate moieties, promote non-specific interactions with the polymer matrix. These adventitious bindings, primarily mediated by electrostatic and hydrogen-bonding interactions, generate a high background signal that obscures the specific recognition signal from the imprinted cavities [27,45]. Consequently, the separation selectivity is significantly compromised, a challenge that becomes particularly critical when working with complex biological samples containing numerous interfering proteins and substances.

The fundamental issue underlying these challenges is the inherent mismatch between the delicate, heterogeneous, and complex nature of glycoproteins and the chemically simplistic nature of traditional MIPs. Therefore, the development of innovative imprinting strategies tailored to the unique structural characteristics of glycoproteins is the key to overcoming these bottlenecks.

## 3. Advanced Strategies for Glycoprotein-Imprinting

To overcome the inherent challenges of glycoprotein imprinting, research has moved beyond traditional bulk imprinting towards more sophisticated and effective strategies. These can be categorized into three main approaches, oriented surface imprinting strategy [46], epitope imprinting strategy [47], and PIM strategy [48], each leveraging distinct mechanisms to tackle the structural and physicochemical complexities of glycoproteins. Critically, the selection and design of these strategies are profoundly influenced by the unique attributes of glycoproteins—namely, their composite structure of a polypeptide backbone with covalently attached glycan chains, and their need to be recognized in native, physiological environments.

### 3.1. Oriented Surface Imprinting

Oriented surface imprinting is a technique that constructs molecular recognition sites exclusively on the surface of a solid support, thereby preventing deep template embedding and facilitating mass transfer [46]. The concept was first introduced by Haupt et al. [49], who used theophylline-imprinted silica gel to produce porous polymer particles with cavities located only on the surface after template removal. With subsequent scientific and technological progress, this approach has gained popularity in protein imprinting and has been progressively applied to the imprinting and separation of glycoproteins [50].

**Figure 2:**
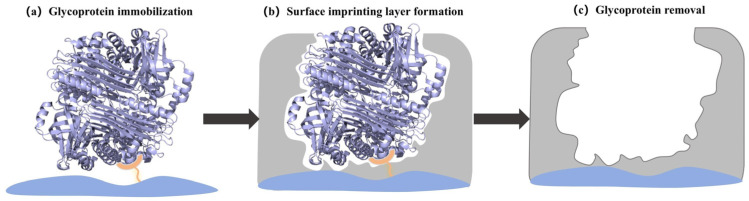
Schematic illustration of the glycoprotein oriented surface imprinting process. Reproduced with permission [46]. Copyright 2020, American Chemical Society.

The oriented surface imprinting of glycoproteins generally involves three key steps, as illustrated in Figure 2 [46]: (a) oriented immobilization of the template, (b) formation of a surface imprinting layer, and (c) removal of the template. In this process, the glycoprotein is first directionally immobilized onto a substrate, followed by the formation of a thin polymer layer around it. After template removal, specific cavities complementary to the template remain on the surface. These cavities can efficiently recognize target glycoproteins from complex samples through intermolecular interactions such as hydrogen bonding, electrostatic forces, or covalent bonding.

Compared to traditional bulk imprinting, the oriented surface imprinting strategy offers the advantages of uniformly distributed binding sites, low mass transfer resistance, easy template elution, and high material reusability [46]. This design effectively addresses the limitations of traditional methods, such as deeply embedded templates and low recognition efficiency, providing robust technical support for the highly specific recognition and separation of glycoproteins and demonstrating broad application potential in bioanalysis and biomedicine [46].

#### 3.1.1. Oriented Immobilization of the Template

In glycoprotein imprinting, the critical step is the oriented surface immobilization of glycoproteins, which is used to anchor the templates in a controlled orientation to preserve the native conformation, thereby ensuring the structural and functional accuracy of the resulting imprinted cavities [46,51]. By far, the boronic acid affinity interaction, lectin-specific recognition, and metal coordination have emerged as the predominant strategies, each offering distinct mechanisms for achieving precise template orientation.

**Figure 3:**
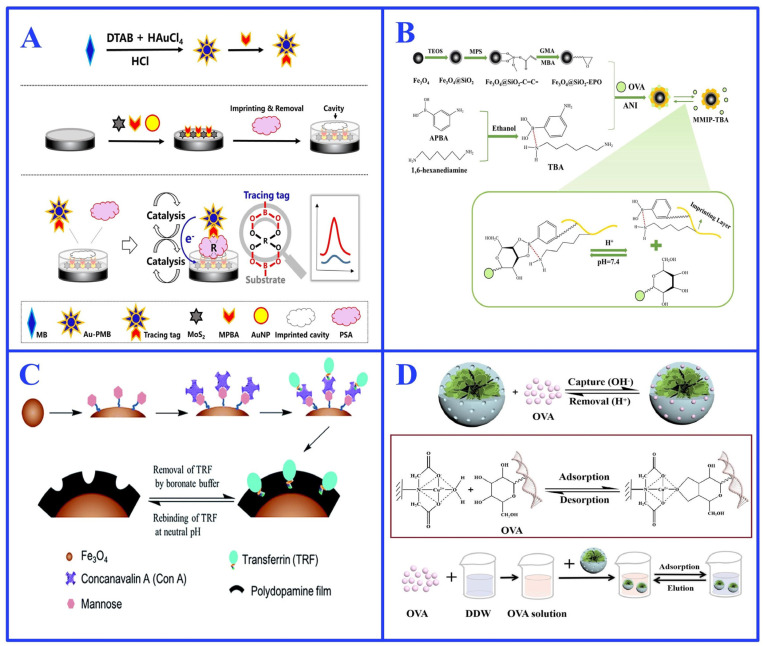
(**A**) Schematic illustration of the boron-affinity surface molecular imprinted biomimetic sensor. Reproduced with permission [54]. Copyright 2017, Springer Nature; (**B**) Schematic illustration of the preparation route of MMIP-TBA and the recognition mechanism between TBA and OVA. Reproduced with permission [61]. Copyright 2025, Elsevier; (**C**) Schematic illustration of Concanavalin A mediated transferrin-imprinted polymer. Reproduced with permission [65]. Copyright 2016, Elsevier; (**D**) Schematic illustration of Cu^2+^-coordinated directed MIP recognition mechanism toward OVA. Reproduced with permission [68]. Copyright 2021, Elsevier.

Boronate affinity-based oriented immobilization is one of the most promising immobilization strategies in glycoprotein surface imprinting. The boronic acid groups can reversibly bind cis-diol structures in the glycan chains of glycoproteins [52]. When the pH is higher than the pKa of the boronic acid, the boron atom undergoes a sp^2^ to sp^3^ hybridization transition, forming cyclic esters with cis-diol structures, which dissociate reversibly when the pH is lower than the pKa value [53]. The gentle and reversible reaction conditions of the boronate affinity interaction significantly contribute to constructing MIPs for biomolecular recognition. In recent years, by integrating boronate affinity interaction with MIT, researchers have developed various MIPs for glycoprotein recognition. Among these, Liu et al. [54] developed a boronate affinity-mediated controllable oriented surface imprinting strategy (Figure 3A). This method enables the directional immobilization of glycoprotein templates on boronic acid-functionalized substrates, yielding MIPs that exhibit artificial antibody-like characteristics, high selectivity, and practical advantages including facile preparation, cost-effectiveness, and operational stability [54]. Based on this strategy, they engineered glycoprotein-imprinted microwell plates compatible with enzyme-linked immunosorbent assay (ELISA) formats, demonstrating high precision in hepatocellular carcinoma diagnosis [55]. In addition, Hou et al. [56] created a molecularly imprinted electrochemical platform for ovalbumin (OVA) detection based on polyethylenimine and boronate affinity, achieving exceptional sensitivity in monitoring allergenic glycoproteins. Zhang et al. [57] developed a boronate-affinity surface-imprinted biomimetic sensor based on a synergistic signal amplification strategy, and achieved ultrasensitive detection of prostate-specific antigen (PSA) with a wide linear range from 1.0 × 10^−4^ to 1.0 × 10^4^ ng/mL and a detection limit of 0.03 pg/mL, significantly advancing the detection of PSA. All the results demonstrate the promising application of the boronate affinity-based oriented immobilization strategy in glycoprotein surface imprinting.

As previously mentioned, boronic acid groups can reversibly bind with cis-diol structures in a pH-dependent manner, with the binding efficiency governed by the pKa value of the specific boronic acid derivative. However, conventional boronic acid functional monomers typically exhibit relatively high pKa values (8.0–9.0), requiring alkaline conditions to achieve efficient binding with cis-diol moieties [58,59]. This alkaline dependency poses significant challenges for glycoprotein imprinting, as non-physiological pH conditions can disrupt the structural integrity and biological functionality of glycoprotein templates [59]. The fundamental incompatibility between optimal boronic acid binding conditions and the preservation of native protein conformations has motivated the development of advanced boronic acid monomers with rationally engineered pKa characteristics.

To overcome this limitation, two principal approaches have been established, the chemical modification and the tandem boronic acid (TBA) strategy. The chemical modification always relates to the introduction of electron-withdrawing groups or the construction of intramolecular B-N/B-O bonds in boronic acid molecules [58,60]. However, the chemical modification of the boronic acid compounds typically requires complicated and challenging synthesis and purification procedures. To address these limitations, the TBA strategy is proposed as a more convenient and versatile strategy, since it relies on the formation of intermolecular B-N coordination through self-assembly between commercial boronic acids and amine compounds. Zhang et al. [61] used this approach with 3-aminophenylboronic acid and 1,6-hexanediamine to create a MIP that operated effectively at pH 7.4, showing high adsorption capacity (201 mg/g) and specificity (IF = 5.74) for OVA (Figure 3B). Similarly, Gong et al. [62] exploited the same strategy to develop a magnetic MIP-based biomimetic sensor, which achieved highly specific (IF = 4.52), sensitive (LOD: 13 pM), and rapid (20 min equilibrium) detection of OVA at physiological pH.

Following boronate affinity, the lectin-based recognition constitutes another important strategy for the oriented immobilization of glycoproteins. Lectins such as concanavalin A (Con A) specifically bind to terminal α-D-mannose and α-D-glucose residues on N-glycans, while wheat germ agglutinin shows affinity for N acetylglucosamine and sialic acid [63,64]. By covalently conjugating lectins to solid supports, target glycoproteins can be efficiently enriched and anchored via their carbohydrate moieties under mild, physiological conditions. For instance, Xue et al. [65] prepared transferrin-imprinted polymer on modified magnetic nanoparticles through Con A-mediated oriented immobilization, enabling specific recognition under physiological conditions without compromising protein activity (Figure 3C). This demonstrates the advantage of lectin-mediated strategies in improving the recognition performance of glycoprotein-imprinted materials.

In addition to lectin-specific recognition, metal coordination serves as another powerful and commonly used technique for the oriented immobilization of glycoproteins in surface molecular imprinting. The immobilized metal ions (e.g., Cu^2+^, Ni^2+^) can specifically coordinate with particular sites on the protein surface (e.g., histidine tags), generating a stable five- or six-coordination rings to precisely anchor the template molecules [66]. This highly specific coordination interaction not only contributes to stabilizing template conformation during imprinting, but also guides the oriented polymerization of functional monomers on the template surface, thereby creating stable imprinted cavities with a high degree of spatial and functional matching to the target glycoprotein [66,67]. Guo et al. [68] developed an OVA MIP onto a Mobil Composition of Matter-48 matrix, based on the Cu^2+^ coordination interaction immobilization (Figure 3D). The resulting MIP achieved a high adsorption capacity of 395.30 mg/g, significantly surpassing that of traditional surface-imprinted polymers and underscoring the advantage of metal coordination in enhancing MIP performance.

#### 3.1.2. Surface Imprinting Layer

In surface imprinting, the thickness of the imprinting layer serves as a critical parameter governing the recognition accuracy and binding performance of MIPs [69]. The optimal thickness is template-dependent, requiring a balance between preventing excessive embedding of template molecules and maintaining the structural integrity of the imprinting sites [70]. Generally, the recommended thickness ranges from one-third to two-thirds of the template molecular size [70]. Achieving this precise thickness control relies on the selection of appropriate materials and fabrication techniques. Commonly used surface imprinting layers, classified by monomer type, include acrylic polymer layers, silica imprinting layers, polydopamine imprinting layers, and phenylboronic acid-functionalized imprinting layers [71,72,73].

Acrylic monomers represent one of the most classical and versatile materials for constructing molecularly imprinted layers. These imprinted layers are constructed through free radical polymerization of acrylic derivatives in the presence of crosslinking agents forming a three-dimensional polymeric network on the substrate surface. The acrylic derivatives mainly include methacrylic acid (MAA) [74], acrylamide (AM) [75], 2-hydroxyethyl methacrylate (HEMA) [76], and N-isopropylacrylamide (NIPAM) [77]. In contrast, the crosslinking agents can be ethylene glycol dimethacrylate (EGDMA) and N,N’-methylenebisacrylamide (MBA), etc. [78,79]. The stability of the imprinting cavities is significantly enhanced by various non-covalent interactions between acrylic monomers and glycoprotein templates. The key advantage of acrylic systems lies in its great versatility in monomer selection and interaction design, coupled with the ability to perform polymerization under ambient conditions in aqueous media, which collectively helps preserve the native conformation of glycoprotein templates.

**Figure 4:**
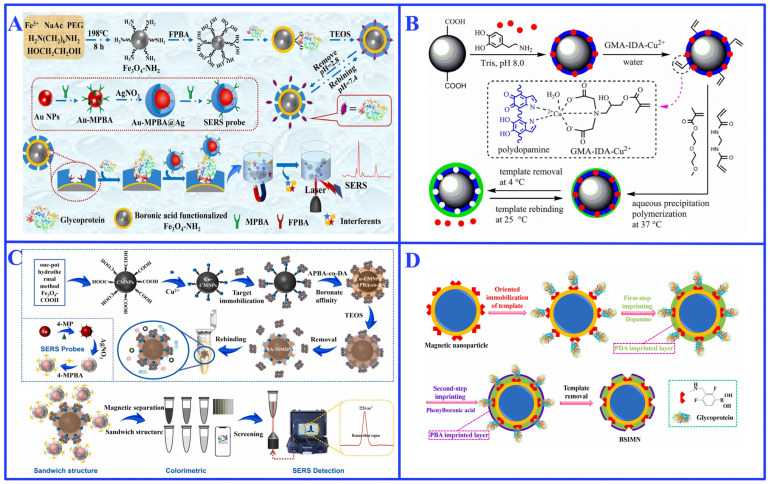
(**A**) Schematic illustration of the preparation and application of a SERS-based silicon-layer boronate affinity biomimetic sensor. Reproduced with permission [81]. Copyright 2022, Elsevier; (**B**) Schematic illustration of the preparation of hollow surface-imprinted microspheres based on the dopamine layer. Reproduced with permission [84]. Copyright 2019, Elsevier; (**C**) Schematic illustration of the double-layer controllable surface-imprinted nanomaterials for detecting glycoprotein biomarkers. Reproduced with permission [87]. Copyright 2025, Elsevier; (**D**) Schematic illustration of the preparation of sequential surface-imprinted magnetic nanoreceptors based on DA and APBA layers. Reproduced with permission [88]. Copyright 2022, Elsevier.

While acrylic monomers offer great versatility, they can present challenges in achieving precise control over the imprinting layer thickness. In contrast, alternative materials offer more straightforward pathways to achieve the recommended dimensional precision. Silica imprinting layers are synthesized via a sol–gel process using tetraethyl orthosilicate (TEOS) as the precursor. Under alkaline catalysis, TEOS undergoes hydrolysis and condensation to form an inorganic-organic hybrid network with a Si–O–Si framework (Figure 4A) [80,81]. Polydopamine (PDA) imprinting layers are produced by dopamine (DA) self-polymerization under alkaline conditions, generating a uniform polydopamine film on the substrate surface (Figure 4B) [82,83]. Both types of imprinting layers exhibit excellent biocompatibility and chemical stability. Importantly, their thickness can be precisely regulated by controlling the reaction time, making them particularly suitable for surface imprinting systems requiring precise structural control [84].

Phenylboronic acid-functionalized imprinting layers offer a targeted strategy for glycoprotein recognition by specifically interacting with cis-diol motifs in glycan structures [73]. These systems function through reversible covalent complexation between boronic acid groups and cis-diols, enabling highly selective template incorporation [73]. When fabricated with functional monomers such as 3-aminophenylboronic acid (APBA), the resulting imprinted interfaces demonstrate preferential affinity for carbohydrate moieties [85], thereby minimizing nonspecific binding to polypeptide regions. This molecular discrimination capability makes such platforms particularly advantageous for glycoprotein assays where glycoform variation serves as a primary diagnostic indicator.

Moreover, boronic acid-functionalized layers exhibit synergistic effects when integrated with polydopamine-based imprinting systems. The complementary characteristics—specifically, the universal surface adhesion capability of polydopamine and the selective molecular recognition properties of phenylboronic acid—create a cooperative architecture that significantly enhances both binding efficiency and imprinting performance. For instance, Zhao et al. [86] developed a sequential imprinting process involving an initial dopamine self-polymerization to create a primary scaffold and a subsequent incorporation of boronic acid ligands (e.g., APBA), which resulted in nanoreceptors with an order-of-magnitude improvement in binding affinity (*K*_a_ = 4.8 × 10^6^ L/mol for DNase I). Similarly, Wang et al. [87] developed a bilayer amphiphilic-controllable MIP (BA-MMIP) by sequentially integrating a poly(APBA-co-DA) imprinting layer with a SiO_2_ barrier to construct a portable SERS-colorimetric dual-mode biomimetic sensor, which suppressed non-specific binding and enabled ultrasensitive transferrin detection at 10.2 pg/mL (Figure 4C). Furthermore, Lang et al. [88] engineered a self-healing magnetic nanoreceptor by integrating boronate-affinity-oriented template immobilization with a sequential PDA/PBA imprinting process, achieving homogeneous imprinted cavities and exceptional selectivity for OVA with a high imprinting factor of 10.1 (Figure 4D). These studies demonstrate that this strategic combination establishes a robust platform for advanced glycoprotein detection and separation applications.

#### 3.1.3. Functional Substrates

Beyond the design of the imprinting layer and the oriented immobilization strategy, the selection of functional substrates is equally critical in glycoprotein-oriented surface imprinting technology. Ideally, substrates should exhibit good biocompatibility, high surface area, and ease of functionalization to achieve efficient template immobilization and precise construction of imprinting sites [89]. Commonly used substrates include silica-based materials, magnetic nanoparticles, carbon materials, noble metals, metal–organic frameworks (MOFs), and their composite nanomaterials [90,91,92].

Silica-based materials (e.g., mesoporous SiO_2_ and SiO_2_ nanospheres) are classical substrates valued for their high stability and rich surface chemistry, as well as good biocompatibility and high specific surface [93]. The abundant surface hydroxyl groups allow for modification with silane coupling agents (e.g., APTES, MPS), introducing functional groups such as amino and thiol moieties that provide specific binding sites for glycoprotein immobilization [93]. For instance, He et al. [70] grafted polyethylene polyamine and boronic acid molecules onto silica nanoparticles to prepare OVA-imprinted nanoparticles. These nanoparticles demonstrated rapid adsorption kinetics, excellent selectivity, reusability, and achieved a high imprinting factor of 4.82 with an adsorption capacity of 243.4 mg/g. Similarly, Li et al. [94] developed boronate affinity-based molecularly imprinted silica nanoparticles by covalently immobilizing a glycoprotein template onto boronic acid-functionalized silica nanoparticles. The resulting MIPs exhibited excellent specificity toward transferrin, characterized by a high binding affinity ((0.71 ± 0.06) × 10^−7^ mol/L) and effective binding at a low pH of 5.0.

**Figure 5:**
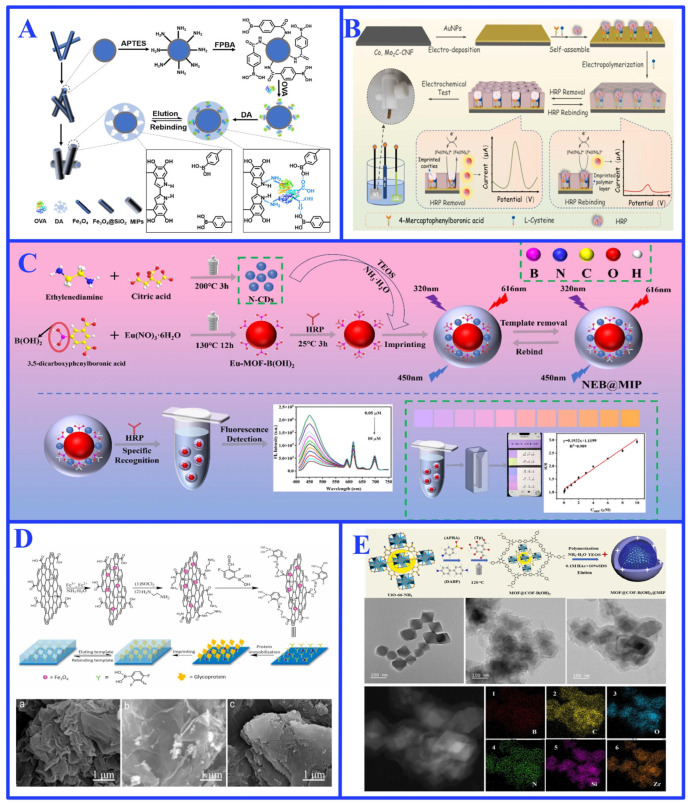
(**A**) Schematic illustration of the preparation process of Fe_3_O_4_@SiO_2_/MIPs. Reproduced with permission [96]. Copyright 2022, Elsevier; (**B**) Schematic illustration of an electrospun carbon fiber-based molecularly imprinted electrochemical biomimetic sensor. Reproduced with permission [100]. Copyright 2023, Elsevier; (**C**) Schematic illustration of a boronic acid-functionalized Eu-MOF fluorescence imprinting biomimetic sensor. Reproduced with permission [104]. Copyright 2025, Springer Nature; (**D**) Schematic illustration of surface molecularly imprinted polymers based on magnetic graphene oxide. Reproduced with permission [108]. Copyright 2021, Elsevier; (**E**) Schematic illustration of a fluorescence-electrochemical imprinting biomimetic sensor platform constructed using MOF@COF core–shell composites. Reproduced with permission [110]. Copyright 2024, Elsevier.

Magnetic materials (e.g., Fe_3_O_4_ nanoparticles) display special advantages in glycoprotein recognition, owing to their superparamagnetic properties and surface modifiability [95]. The Fe_3_O_4_@SiO_2_/MIPs prepared through surface modification not only maintain high adsorption performance but also enable rapid magnetic separation and recovery [96]. Furthermore, the SiO_2_ layer helps prevent the oxidation and aggregation of Fe_3_O_4_ nanoparticles, thereby improving their stability and biocompatibility while providing abundant sites for further surface modification [97]. For example, Guo et al. [96] employed a pre-immobilized surface imprinting technique on surface-modified Fe_3_O_4_ nanorods to prepare an OVA-imprinted polymer (Figure 5A). This MIP exhibited a high adsorption capacity (175.2 mg/g) and a fast mass transfer rate (equilibrium within 40 min), effectively combining the benefits of magnetic separation with the specific recognition of MIPs. In a detection application, Xu et al. [98] developed a magnetic molecularly imprinted system using boronic acid-functionalized magnetic nanoparticles as substrates. By integrating boronate-affinity MIPs with boric acid-functionalized rod-like fluorescent porphyrin aggregates into a sandwich structure, they constructed a biomimetic fluorescent sensor for transferrin with sensitive detection capabilities. The system achieved recoveries between 97.4% and 104.4%, a linear range of 2–50 μg/mL, and a detection limit of 0.74 μg/mL.

Carbon materials (e.g., carbon nanofibers, graphene, carbon nanotubes, mesoporous carbon and biomass-activated carbon) have attracted considerable attention due to their high mechanical strength, chemical stability and large specific surface area [99]. Chen et al. [100] developed a self-supporting electrochemical biomimetic sensor based on electrospun carbon nanofibers (CNFs) (Figure 5B). By precisely electropolymerizing an ultrathin MIP layer on the CNF electrode, they achieved a high specificity and excellent selective recognition efficiency toward horseradish peroxidase, demonstrating the advantage of electropolymerization for achieving conformal, intimate contact between the MIP recognition layer and the conductive substrate, with detection limit of 7.4 fg/mL and recoveries ranging from 89.21% to 116.68%. In another work, Ding et al. [101] fabricated a novel imprinted polymer using biomass-activated carbon as a carrier, which exhibited a high binding capacity of 196.2 mg/g, rapid adsorption dynamics of 40 min equilibrium, excellent selectivity and satisfactory reusability for OVA.

Noble metal nanomaterials (e.g., Au, Ag) provide ideal platforms for highly sensitive detection due to their unique electronic structures and surface plasmon resonance effects [102]. Geng et al. [91] constructed a sandwich-type surface-enhanced Raman scattering biomimetic sensor for ultrasensitive detection of transferrin by integrating boronic acid-functionalized magnetic MIPs with 4-mercaptophenylboronic acid-modified gold-silver core–shell nanostructures. The proposed biomimetic sensor achieved an exceptionally low detection limit of 0.004 ng/mL and exhibited satisfactory performance in detecting transferrin in spiked serum samples, demonstrating great potential for clinical diagnostics.

MOFs, known for their tunable pore structures and enormous specific surface areas, offer new possibilities for constructing high-density imprinting sites [92]. Gong et al. [103] developed MIP nanoparticles based on a MOF (MIL-101(Cr)-NH_2_) loaded with high-density boric acid for specific OVA enrichment. The large specific surface area of MOF skeleton contributed to a high functional group density (4.66%), and the resulting MIP achieved a high adsorption capacity of 482.56 mg/g, reached dynamic equilibrium in 20 min, and exhibited an imprinting factor of up to 5.3. Ma et al. [104] constructed a smartphone-based molecularly imprinted ratiometric fluorescence biomimetic sensor using boronic acid-functionalized metal–organic frameworks (Eu-MOF-B(OH)_2_) as the support (Figure 5C). The high surface area of the Eu-MOF-B(OH)_2_ favored covalent binding with the target glycoprotein horseradish peroxidase. The obtained biomimetic sensor achieved a detection limit of 0.01 µM and a satisfactory recovery of 92.0–98.5% in human urine and human serum.

In recent years, composite nanomaterials have emerged as a new research direction by synergistically integrating the advantageous characteristics of different components [105,106,107]. For instance, magnetic-graphene composites combine the magnetic responsiveness of Fe_3_O_4_ with the high specific surface area of graphene oxide, thereby enhancing both the separation efficiency and binding capacity for glycoproteins (Figure 5D) [108,109]. Furthermore, MOF-COF core–shell structures not only maintain high porosity from both components but also overcome aggregation-caused quenching effects, providing new avenues for developing fluorescent glycoprotein biomimetic sensors with improved sensitivity (Figure 5E) [110].

The progression from classical silica to sophisticated composites underscores the critical role of substrate engineering. The rational design provides tailored platforms for template orientation and polymer growth, ultimately enabling the creation of MIPs with superior specificity, faster binding kinetics, and enhanced utility in complex biological matrices.

#### 3.1.4. Challenges and Limitations

Notwithstanding the remarkable progress, oriented surface imprinting for glycoproteins still confronts several entrenched challenges that warrant further investigation. The precision offered by advanced immobilization techniques (e.g., boronate affinity, metal coordination) inherently restricts the spectrum of addressable templates, as each method necessitates specific structural handles on the target. Therefore, broadening the repertoire of such gentle and efficient oriented-immobilization methodologies constitutes a persistent objective within this strategic framework. Even for promising strategies that successfully address specific issues, such as the TBA complex for operation under physiological pH, new questions regarding their long-term stability under repetitive operational cycles remain open and necessitate dedicated study.

Moreover, the imperative to achieve a thin, well-defined imprinting layer for optimal performance introduces a potential compromise with mechanical stability and binding-site integrity, particularly under conditions of repeated use or in complex matrices. Future developments within this paradigm should therefore prioritize the design of interfacial layers that effectively reconcile the requisite structural precision with sustained mechanical and chemical robustness.

A further and more fundamental challenge arises from the inherent tension between achieving high-fidelity imprinting and maintaining physiological compatibility during fabrication. Polymerization conditions optimized for cavity formation and stability often involve organic solvents or non-physiological pH, potentially compromising the native conformation of the glycoprotein template. Bridging this gap to develop robust imprinting protocols under fully aqueous, physiologically relevant conditions remains a significant hurdle for ensuring the biological relevance of the resulting MIPs.

### 3.2. Epitope Imprinting

**Figure 6:**
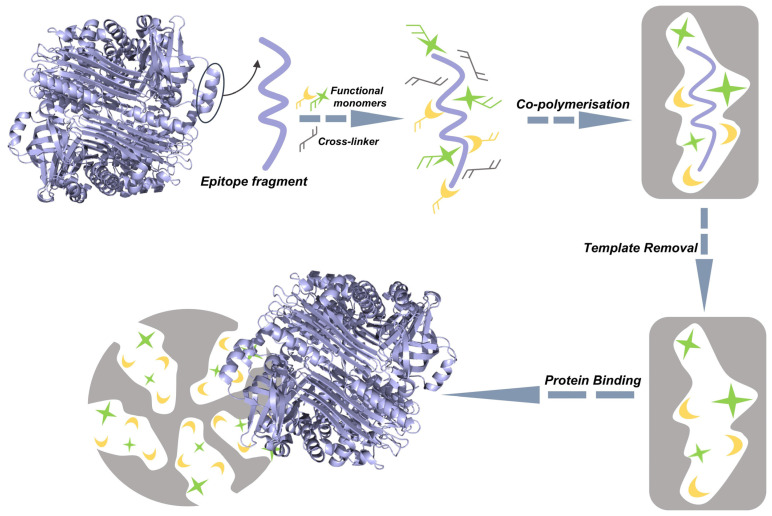
Schematic illustration of the epitope imprinting concept. Reproduced with permission [120]. Copyright 2021, The American Association for the Advancement of Science.

While oriented surface imprinting offers significant advancements, fundamental challenges of glycoprotein imprinting—such as structural instability, inefficient template removal, and prominent nonspecific binding—are not fully resolved when using the intact glycoprotein as a template [44,111]. In response, epitope imprinting has emerged as a powerful alternative strategy. This approach, originally pioneered by Rachkov and Minoura [112,113], utilizes short, characteristic segments of the target as synthetic templates, thereby bypassing the need for the full-length protein, as illustrated in Figure 6 [114,115]. The selected epitopes offer inherent advantages of structural simplicity and cost-effective production, which substantially reduce system complexity while maintaining molecular specificity [115].

Building upon this template design, the epitope strategy enables more precise engineering of the binding site architecture. This precision confers distinct advantages for analysis in complex matrices, delivering an optimal combination of high recognition specificity and enhanced analytical robustness [116,117]. Consequently, epitope imprinting has become particularly valuable in biomarker detection and diagnostic applications, effectively overcoming the fundamental limitations of conventional whole-protein imprinting [118,119].

Based on the number and type of epitopes selected, epitope imprinting strategies are systematically classified into single-epitope and multi-epitope imprinting.

#### 3.2.1. Single-Epitope Imprinting

As the fundamental form of epitope imprinting, the single-epitope approach creates well-defined cavities using only one characteristic peptide or glycan segment [121]. This strategy maintains high specificity while offering maximal operational simplicity.

The selection of an appropriate epitope is the cornerstone of this method, directly governing the specificity and affinity of the resulting MIPs. Ideal epitopes must exhibit structural uniqueness to ensure target discrimination and maintain a stable conformation throughout the polymerization process [122]. Commonly adopted epitopes include terminal sequences of glycoproteins, immunodominant peptide fragments, or disease-specific glycan motifs [123].

**Figure 7:**
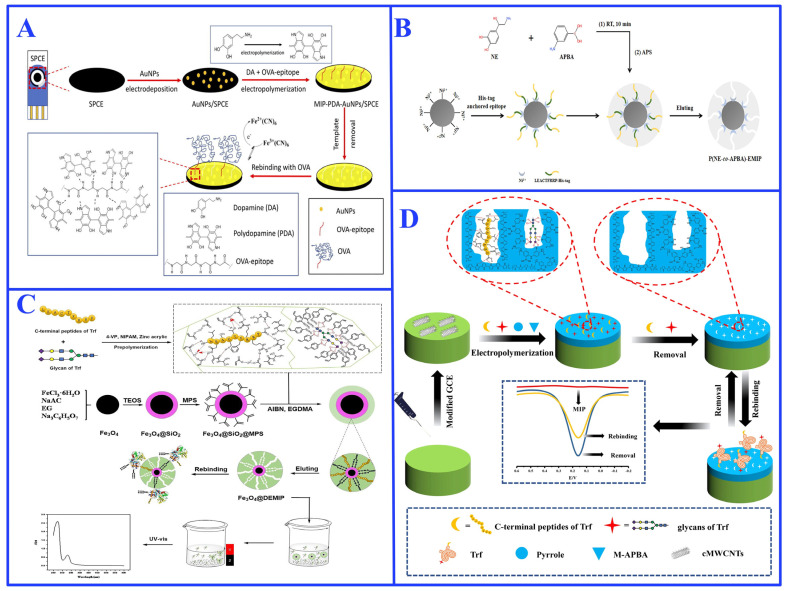
(**A**) Schematic illustration of an electrochemical biomimetic sensor prepared based on immunoglobulin E-binding epitope imprinting. Reproduced with permission [83]. Copyright 2021, Elsevier; (**B**) Schematic illustration of transferrin single-epitope imprint polymer. Reproduced with permission [124]. Copyright 2024, Elsevier; (**C**) Schematic illustration of dual-site imprinted polymer for the cooperative recognition of transferrin Reproduced with permission [129]. Copyright 2024, Elsevier; (**D**) Schematic illustration of an enzyme-free electrochemical biomimetic sensor prepared using a mixture of glycoprotein epitopes. Reproduced with permission [130]. Copyright 2023, Springer Nature.

The small size and structural stability of epitope templates facilitate efficient template removal and faster mass transfer kinetics compared to whole-protein imprinting [120]. The reduced system complexity also minimizes nonspecific binding and enhances reproducibility. For instance, Khumsap et al. [83] used the immunoglobulin E-binding epitope as the template to construct a MIP based electrochemical platform, the platform detected OVA sensitively and efficiently, with linear range from 23.25 to 232.50 nM (1 to 10 ppm), limit of detection (LOD) of 10.76 nM (0.46 ppm), and limit of quantification (LOQ) of 35.87 nM (1.54 ppm) (Figure 7A). Wang et al. [124] employed Ni^2+^-His-tag chelation to directionally immobilize a peptide sequence of transferrin (Figure 7B), resulting in a molecularly imprinted polymer that exhibited excellent recognition for the target protein, with an adsorption capacity of 94.2 mg/g and an imprinting factor of 3.50. Similarly, Xu et al. [125] anchored the N-terminal epitope of CD59 protein using host-guest pair cucurbit[7]uril and l-phenylalanine. The resulting MIP demonstrated a specific adsorption capacity of 88.2 mg/g toward CD59, an imprinting factor of 5.63, high reusability, and was successfully applied to detect CD59 in human serum with a detection limit of 0.44 ng/mL and recoveries ranging from 82.5% to 104.8%.

Through rational epitope design and optimized imprinting conditions, single-epitope imprinting provides a robust platform for specific glycoprotein recognition, advancing applications in bioseparation, clinical diagnostics, and biomarker discovery.

#### 3.2.2. Multi-Epitope Imprinting

Building upon single-epitope imprinting, the multi-epitope strategy employs multiple complementary epitopes as co-templates to create synergistic recognition interfaces. The cooperative effects generated by interconnected binding sites enhance detection sensitivity, broaden specificity across protein isoforms, and improve robustness in complex biological environments [126,127].

This strategy is particularly advantageous for glycoproteins with heterogeneous and complex surface features. A representative example is transferrin, which possesses multiple oligosaccharide branches alongside various exposed polypeptide segments [128]. A powerful implementation of this approach—known specifically as hybrid epitope imprinting—strategically combines both peptide and glycan epitopes as hybrid templates [129,130]. This dual-targeting approach leverages the precise binding capabilities of peptide sequences alongside the distinctive recognition properties of carbohydrate motifs, enabling simultaneous engagement with different structural regions of a glycoprotein. For instance, He et al. [129] used such a hybrid imprinting system to develop a dual-affinity MIP using both C-terminal nonapeptides and transferrin glycans as epitope templates, leveraging metal chelation and boronate affinity interactions (Figure 7C). The resulting MIP for transferrin achieved rapid magnetic separation of 60 s and fast adsorption equilibrium of 100 min, a high adsorption capacity of 43.96 mg/g, and an imprinting factor of 6.60. In subsequent work [130], the same group constructed a MIP-based electrochemical biomimetic sensor using similar dual-epitope recognition (i.e., hybrid recognition strategy), achieving a wide linear range (0.125–1.25 μM) and low detection limit (0.024 μM) for transferrin detection in spiked human serum, with recoveries ranging from 94.7% to 106.0% (Figure 7D).

The synergistic effects and flexible template design of multi-epitope imprinting enable superior recognition accuracy, enhanced binding affinity, and improved discrimination against non-target proteins. These capabilities make the strategy particularly valuable for precise analysis of complex glycoprotein variants and promising for advancing specific disease biomarker detection and robust diagnostic platform development.

#### 3.2.3. Challenges and Limitations

The epitope imprinting strategy, while circumventing many issues associated with whole-protein templates, faces its own set of bottlenecks, chief among which is the rational selection and efficacy of the epitope itself. Current epitope choice often relies on empirical knowledge or mimics of natural antibodies, lacking universal principles for predictive design [117,123]. A significant risk is “conformational mismatch”, wherein the solution-phase conformation of a linear peptide epitope deviates from its native spatial structure within the intact protein, resulting in imprinted cavities with reduced affinity for the full-length target and compromised recognition efficiency [120]. Furthermore, the optimal spatial arrangement of multiple epitope sites within the polymer matrix remains a significant and underexplored challenge. Additionally, for glycan-based epitopes, their inherent microheterogeneity makes obtaining structurally uniform oligosaccharide templates challenging and costly [131]. These constraints currently limit the application of epitope-imprinted polymers in scenarios demanding ultra-high-resolution recognition of specific glycosylation variants. The pursuit of enhanced functionality beyond structural mimicry constitutes another strategic frontier, leading to the approach of PIM.

### 3.3. Post-Imprinting Modification

**Figure 8:**
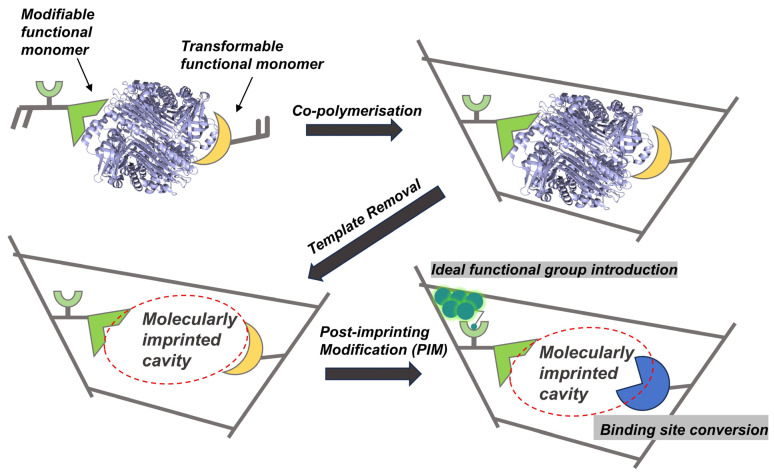
Schematic illustration of PIM principles. Reproduced with permission [133]. Copyright 2018, Royal Society of Chemistry.

PIM is a technique inspired by protein post-translational modifications (PTMs) in biology [132], which involves introducing specific chemical functionalizations into pre-formed MIPs after template removal (Figure 8) [133]. By directionally altering or introducing functional groups, PIM enhances the recognition properties of MIPs while preserving their predetermined cavity structures [134]. This strategy not only effectively safeguards the recognition capability of the imprinted cavity but also endows the polymer with new functions, demonstrating exceptional flexibility in tailoring binding properties and remarkable versatility in signal transduction [48]. Consequently, PIM opens new application dimensions for glycoprotein MIPs in signal response and visual detection.

Based on the primary modification objectives, PIM strategies are primarily divided into two fundamental categories: binding site conversion and ideal functional group introduction [48]. The binding site conversion strategy reconstructs functional groups within the imprinted cavities to introduce high-affinity binding sites, thereby improving the selective recognition capability of the polymers toward target molecules [135,136]. A prominent demonstration of this strategy is the post-imprinting incorporation of phenyl-Schiff base groups into the cavities of a sialic acid-imprinted magnetic polymer. This approach established a dual-capture system that exploited dynamic imine bond formation to markedly enhance both the affinity and specificity for sialic acid and sialylated glycoproteins, thereby demonstrating how this strategy enhances selectivity for applications in separation science [136].

**Figure 9:**
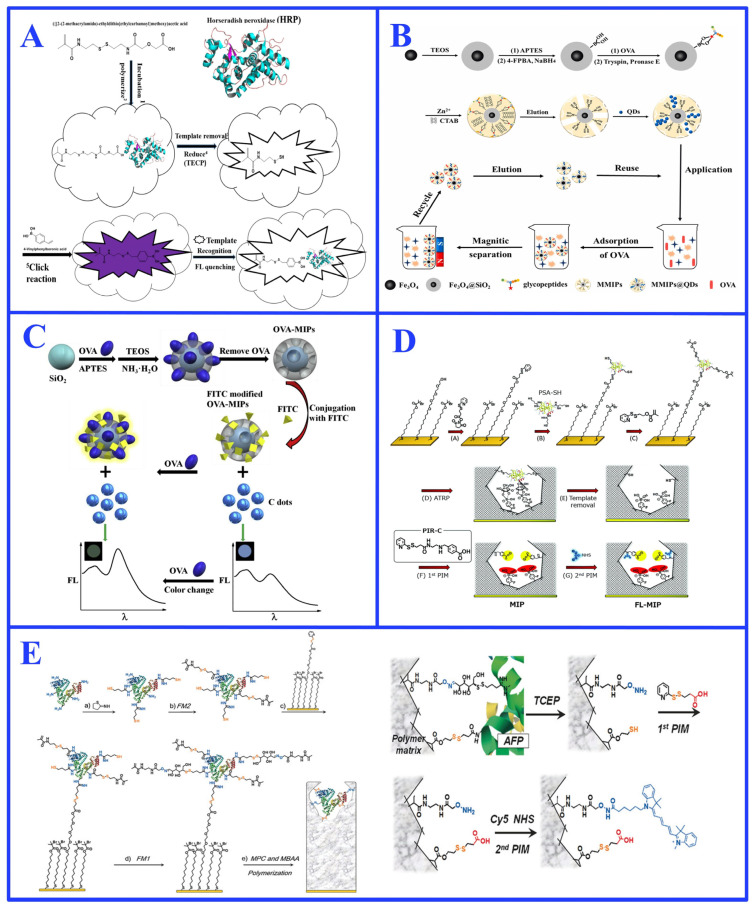
(**A**) Schematic illustration of converting horseradish peroxidase recognition to a fluorescent signal through one-step post-labeling modification. Reproduced with permission [138]. Copyright 2017, Elsevier; (**B**) Schematic illustration of a fluorescent biomimetic sensor for efficient detection of OVA based on PIM. Reproduced with permission [26]. Copyright 2022, Elsevier; (**C**) Schematic illustration of designing fluorescent biomimetic nanosensors for OVA detection through post-modification. Reproduced with permission [139]. Copyright 2020, Elsevier; (**D**) Schematic illustration of nano-cavities with multifunctional groups prepared by PIM for the detection of prostate cancer biomarkers. Reproduced with permission [140]. Copyright 2020, Royal Society of Chemistry; (**E**) Schematic illustration of a programmable signal molecule-recognizing nanocavity prepared by post-modification after two-step imprinting. Reproduced with permission [141]. Copyright 2016, Wiley.

The ideal functional group introduction strategy incorporates novel functional units to endow the MIP with new capabilities, particularly signal transduction for integrated “recognition–detection” functionality, which is pivotal for biomimetic sensing and diagnostic applications [137,138]. This is commonly achieved by introducing signaling units such as fluorescent molecules or quantum dots into the imprinted cavities (Figure 9A–C) [26,138,139]. This concept is exemplified by the covalent grafting of nitrogen-doped graphene quantum dots (QDs) via PIM onto glycopeptide-imprinted magnetic polymers, which resulted in a fluorescent biomimetic sensor (MMIPs@QDs) capable of the highly sensitive and selective detection of OVA in complex egg white samples (Figure 9B) [26]. This strategy can be extended through sophisticated multi-step procedures to create highly complex and programmable systems (as exemplified in Figure 9D,E) [140,141]. For instance, using two cleavable functional monomers followed by independent stepwise PIMs enabled the precise introduction of a carboxylate recognition site and a fluorescent Cy5 dye into α-fetoprotein (AFP)-imprinted cavities, creating a fluorescent-signaling MIP with exceptional sensitivity (Figure 9E) [141]. These works masterfully showcase the unique power of multi-step PIM in engineering complex, multi-analyte detection platforms that are difficult to achieve by conventional imprinting methods. Such PIM-enhanced MIPs serve as robust alternatives to biological receptors in systems such as point-of-care testing devices for viral antigens or cancer biomarkers [140,141].

Beyond these core applications in separation, sensing, and diagnostics, the technology also shows expanding potential in biomedical fields such as targeted drug delivery systems and high-contrast cellular imaging, where tailored surface properties and signaling capabilities prove essential for advanced applications.

However, despite its advantages in functional enhancement, PIM faces several technical limitations that hinder its practical application. The functional reagents required for PIM often involve multi-step organic synthesis, resulting in complex structures and cumbersome modification processes. More critically, PIM reactions are seldom quantitative or perfectly regioselective, leading to random modification sites and non-uniform modification degrees. This heterogeneity risks altering the micro-environment of binding cavities in unpredictable ways, potentially blocking access or degrading recognition performance [48,133]. Furthermore, multi-step PIM procedures significantly increase fabrication complexity and cost, while posing substantial challenges for batch-to-batch reproducibility [142,143]. These practical limitations are compounded by the fundamental incomplete understanding of structure-activity relationships between introduced functional groups and molecular recognition mechanisms.

Fortunately, the emergence of precision PIM techniques, such as those leveraging “click chemistry”, is beginning to address these challenges. These methods enable highly site-specific and quantitative modifications, which minimize the risk of cavity distortion and enhance the reliability of functional integration [138]. This direction aims to achieve modular functionalization while preserving the integrity of the imprinted cavity, representing a pathway toward more controlled and predictable PIM outcomes.

## 4. Comparative Analysis and Strategic Outlook

The three principal strategies detailed above—oriented surface imprinting, epitope imprinting, and PIM—each address the persistent challenges in glycoprotein imprinting through distinct mechanisms. Given their complementary strengths and limitations, a critical and comparative evaluation of their performance is therefore essential to inform rational strategy selection and to identify future research trajectories. Table 1 summarizes their characteristics based on the representative studies reviewed in the preceding sections.

A comparative analysis reveals that oriented surface imprinting generally delivers high adsorption capacities and rapid binding kinetics (as exemplified in Section 3.1), a direct benefit of confining recognition sites to accessible surfaces. This performance profile makes it particularly suitable for preparative-scale separation and enrichment, especially when combined with magnetic supports for facile handling. However, its fundamental constraint is the reliance on specific, pre-existing handles on the template glycoprotein (e.g., glycan cis-diols for boronate affinity, specific carbohydrate motifs for lectin recognition, or polyhistidine tags for metal-ion coordination), which inherently limits generality.

In contrast, epitope imprinting typically achieves efficient template removal and yields high imprinting factors, correlating with exceptional binding specificity and analytical reproducibility (as exemplified in Section 3.2). These attributes are critical for diagnostic analysis and precise quantification in complex biological matrices. The associated trade-offs include a more moderate adsorption capacity for the intact protein and a critical dependence on the judicious selection of an epitope that accurately mirrors its native three-dimensional conformation within the full-length protein. Furthermore, the high specificity achieved for a chosen epitope, particularly a glycan motif, inherently narrows the recognition profile, potentially rendering the MIP ineffective against other glycoforms of the same glycoprotein—a key consideration when the diagnostic target is not a single, defined glycoform.

PIM provides unparalleled functionalization versatility, allowing the post-synthetic introduction of signaling elements or stimuli-responsive groups into pre-formed imprinted cavities (as exemplified in Section 3.3). This capability is pivotal for engineering integrated “recognition–transduction” systems. However, this advantage is counterbalanced by substantially increased synthetic complexity, higher costs, and significant challenges in maintaining batch-to-batch reproducibility, as the modification reactions are often non-quantitative and risk altering the delicate microenvironment of the imprinted site. A more fundamental strategic trade-off arises between introduced functionality and preserved specificity. Newly grafted chemical or signaling moieties can inadvertently compromise specificity by perturbing the cavity, potentially enhancing signal output at the expense of recognition fidelity.

Therefore, a pragmatic selection framework can be proposed. For applications demanding high binding capacity and processing speed, such as large-scale glycoprotein enrichment, oriented surface imprinting is often the most suitable choice. Scenarios requiring the highest degree of specificity and reliability in complex samples, such as clinical biomarker detection, are best served by epitope imprinting. When advanced, built-in functionality—such as intrinsic signal generation or controllable release—is paramount, the PIM approach, often in synergistic combination with a primary imprinting strategy, offers the necessary design flexibility, albeit with greater procedural sophistication.

It is important to acknowledge that the performance metrics summarized in Table 1 and discussed above are aggregated from a diverse body of literature. Apparent variations in reported values—for instance, in adsorption capacity or imprinting factor for nominally similar strategies—often originate from methodological differences rather than contradictions in fundamental principles. These differences include variations in template source and glycosylation pattern, monomer-to-template ratio, cross-linking density, substrate morphology, and specific assay conditions. Such variability underscores the context-dependent nature of MIP performance and highlights the current need for more standardized imprinting and evaluation protocols to enable direct and unambiguous comparisons. Furthermore, a discernible “performance gap” is frequently observed between results obtained in idealized buffer systems and those in complex, undiluted biological matrices such as serum or plasma. This gap, particularly evident in parameters like sensitivity and selectivity, underscores the importance of validating MIP performance in clinically relevant environments. Comprehensive assessment against challenging interferents, along with systematic studies on long-term stability and reusability, is therefore crucial for evaluating practical utility and advancing toward real-world applications.

Looking forward, the complementary strengths of these strategies suggest that significant advances may emerge from their convergent integration. Hybrid systems, for example, could combine the precision of multi-epitope recognition with the operational benefits of surface imprinting, further augmented by targeted PIM to introduce stimuli-responsive or signaling functions, thus yielding sophisticated synthetic receptors with enhanced overall performance. Early implementations of this integrated approach, such as the creation of self-healing nanoreceptors via the integration of boronate-affinity-oriented immobilization with sequential surface imprinting [88], demonstrate its feasibility. This integrated approach represents a promising blueprint for next-generation synthetic receptors with enhanced capabilities.

Concurrently, addressing the practical and environmental considerations inherent in MIP fabrication is becoming increasingly important. Future development could greatly benefit from embracing green chemistry principles, which align with the need for sustainable and clinically translatable technologies [144]. Potential pathways include: (1) exploring sustainable feedstocks, such as monomers and cross-linkers derived from renewable resources; (2) adopting benign synthesis methods, like aqueous-phase polymerization and energy-efficient photo-initiation; (3) developing greener elution and regeneration protocols based on mild, aqueous-compatible stimuli; and (4) designing for extended reusability and, where applicable, exploring biodegradable components. The systematic validation of such approaches against conventional methods will be crucial for their adoption.

Finally, to translate these integrated and sustainable systems from promising concepts into robust technologies, and to reconcile performance variations reported in the literature, the field must prioritize the establishment of standardized benchmarking frameworks. This entails developing consensus protocols for the synthesis, characterization, and reporting of data for glycoprotein-imprinted MIPs. Such standardization is a prerequisite for meaningful meta-analysis, for distinguishing genuine strategic advantages from experimental artifacts, and for providing the reliable comparative data essential for clinical translation and commercial development.

Underpinning all these strategic directions—integration, sustainability, and standardization—are three fundamental challenges that define the frontier of the field. The foremost is conformational fidelity: creating cavities that truly mirror the biologically active state of a glycoprotein, not an artifact of the imprinting process. Next is the paradigm of rational design: supplanting trial-and-error with computational and AI-driven methods to predict and achieve desired affinity and specificity. Finally, the imperative of translational validation: proving efficacy and robustness in clinically relevant environments, which goes beyond analytical figures of merit. Progress in molecular imprinting for glycoproteins will ultimately be measured by the advances made against these intrinsic hurdles, which collectively gate the journey from sophisticated synthetic materials to indispensable biomedical tools.

## 5. Conclusions

MIPs have established themselves as a powerful and versatile platform for the specific recognition of glycoproteins, effectively addressing critical limitations inherent to conventional biological receptors. The innovative strategies discussed in this review, including oriented surface imprinting, epitope imprinting, and PIM, collectively address the core challenges of traditional imprinting, driving a paradigm shift from whole-molecule imprinting to the engineering of stable, accessible, and functionalized interfaces.

The comparative analysis underscores that the strategic value of each approach is application-defined, leading logically to their future integration. Realizing this integrated vision, however, hinges on overcoming the fundamental challenges of conformational fidelity, rational design, and translational validation outlined therein. By tackling these challenges through interdisciplinary convergence, MIPs are poised to transition from powerful in vitro tools to indispensable components in diagnostics, therapeutics, and glycoproteomics.

## Figures and Tables

**Table 1:** Comparative of advanced glycoprotein imprinting strategies *.

Evaluation Criterion	Oriented Surface Imprinting	Epitope Imprinting	Post-Imprinting Modification	Remarks
Imprinting Factor	Moderate–High	Very High	Variable (can be enhanced)	Gold-standard metric for specificity
Adsorption Capacity	High	Low–Moderate	Dependent on the base MIP	Reflects practical loading potential
Binding Kinetics	Fast	Moderate	Kinetics of the base MIP	Critical for throughput and biomimetic sensing speed
Template Removal	Good (surface sites only)	Very High (due to small template)	Good (depends on base MIP)	Impacts MIP preparation ease and final purity
Generality	Low (needs specific handle)	Low–Moderate (needs effective epitope)	High (versatile chemistry)	Applicability to diverse, unmodified targets
Fabrication Complexity	Moderate	Low–Moderate	High	Multi-step synthesis/modification in PIM
Functional Versatility	Limited (by immobilization chemistry)	Limited (by epitope)	Very High (tailorable)	Ease of integrating diverse transduction mechanisms (optical, electrochemical) and stimuli-responsive release triggers
Optimal Application Scenario	High-capacity, high-throughput processing (e.g., preparative enrichment)	High-fidelity analysis in complex matrices (e.g., diagnostic detection)	Function-driven system design (e.g., signal transduction, controllable release)	Primary criterion for rational strategy selection
Key Strength	Capacity, speed, easy integration with functional supports	Specificity, reproducibility, gentle elution	Unmatched ability to engineer functionality post-synthesis	
Key Limitation	Target scope limited by required handles	Performance hinges on epitope choice	Complexity, reproducibility, risk of cavity distortion	

* Reported values are representative ranges compiled from the literature and are highly dependent on specific template, monomer composition, polymer architecture, and measurement conditions. They are intended for qualitative strategic comparison.

## Data Availability

Data available on request from the authors.
